# Recent Advances in the Genetic and Biochemical Mechanisms of Rice Resistance to Brown Planthoppers (*Nilaparvata lugens* Stål)

**DOI:** 10.3390/ijms242316959

**Published:** 2023-11-30

**Authors:** Shaojie Shi, Huiying Wang, Wenjun Zha, Yan Wu, Kai Liu, Deze Xu, Guangcun He, Lei Zhou, Aiqing You

**Affiliations:** 1 Laboratory of Crop Molecular Breeding, Ministry of Agriculture and Rural Affairs, Hubei Key Laboratory of Food Crop Germplasm and Genetic Improvement, Food Crops Institute, Hubei Academy of Agricultural Sciences, Wuhan 430064, China; shishaojie@hbaas.com (S.S.); wanghuiying0321@126.com (H.W.);; 2 State Key Laboratory of Hybrid Rice, College of Life Sciences, Wuhan University, Wuhan 430072, China; 3 Hubei Hongshan Laboratory, Wuhan 430070, China

**Keywords:** rice, brown planthopper, defense responses, BPH-resistance genes, integrated pest management

## Abstract

Rice (*Oryza sativa* L.) is the staple food of more than half of Earth’s population. Brown planthopper (*Nilaparvata lugens* Stål, BPH) is a host-specific pest of rice responsible for inducing major losses in rice production. Utilizing host resistance to control *N. lugens* is considered to be the most cost-effective method. Therefore, the exploration of resistance genes and resistance mechanisms has become the focus of breeders’ attention. During the long-term co-evolution process, rice has evolved multiple mechanisms to defend against BPH infection, and BPHs have evolved various mechanisms to overcome the defenses of rice plants. More than 49 BPH-resistance genes/QTLs have been reported to date, and the responses of rice to BPH feeding activity involve various processes, including MAPK activation, plant hormone production, Ca^2+^ flux, etc. Several secretory proteins of BPHs have been identified and are involved in activating or suppressing a series of defense responses in rice. Here, we review some recent advances in our understanding of rice–BPH interactions. We also discuss research progress in controlling methods of brown planthoppers, including cultural management, trap cropping, and biological control. These studies contribute to the establishment of green integrated management systems for brown planthoppers.

## 1. Introduction

The brown planthopper (*Nilaparvata lugens* Stål, BPH) is a host-specific herbivore that is widespread in Asia, Australia, and the South Pacific islands [1]. *N. lugens* soaks up phloem sap by inserting needle-like stylets into the vascular tissue of rice (*Oryza sativa* L.) [2]. Large amounts of BPHs often gathered in groups to harm plants, and caused wilting, yellowing, and even death of rice plants, as well as “hopperburn” in BPH-susceptible rice fields [3]. BPHs are also vectors of various viruses of rice, such as the grassy stunt virus and ragged stunt virus, which were introduced into rice plants during the *N. lugens* feeding process [4,5,6]. Direct and indirect economic losses induced by BPH feeding in Asia alone exceed hundreds of millions of dollars on an annual basis [7]. Brown planthoppers have become one of the most serious pests that harm rice production [3].

Currently, the application of chemical insecticides remains the major approach to controlling BPH in the field [8]. However, the widespread use of these compounds is hazardous to human health and the environment and has side effects that impact the natural enemies of BPH [9]. In addition, the indiscriminate use of pesticides can promote the emergence of insecticide resistance in BPHs [10,11]. Wu et al. found that the insecticide resistance to different insecticides (including imidacloprid, buprofezin, thiamethoxam, pymetrozine fufprole, chlorpyrifos, sulfoxafor, nitenpyram) of 69 *N. lugens* populations collected from eight Chinese provinces improved to varying degrees [12]. This led to a significant reduction in the toxicity efficiency against BPH [13,14,15,16]. Therefore, other BPH management strategies that are greener, healthier, and more sustainable must be developed. Utilizing the inherent resistance genes of rice to cultivate resistant rice varieties has been widely considered as the most cost-effective method for sustainable BPH control [17,18,19].

DNA sequence data show that the host of BPHs began to gradually transfer from *Leersia* to rice approximately 2.5 million years ago [1]. Rice has since evolved sophisticated defense systems to resist BPH infection, and BPHs have evolved various mechanisms to overcome these defenses [1]. Here, we review recent advances in research on the detection of BPH-resistance genes/QTLs, the mechanisms by which rice resists BPH infestations, and the roles of BPH secretory proteins in activating or suppressing rice defenses, and discuss their utilization in diminishing damage caused by brown planthoppers. Additionally, we discuss the research progress in *N. lugens* controlling methods. The insights from this review will enhance our understanding of the survival competition mechanism between rice and BPH and aid the development of strategies to establish green integrated BPH management.

## 2. BPH-Resistance Gene Mapping

The *indica* cultivar Mudgo, the first BPH-resistant rice germplasm, was identified in 1969 by the International Rice Research Institute [20]. *Bph1*, the first BPH-resistance gene identified from Mudgo, was mapped on chromosome 12 [21]. In recent decades, more than 49 BPH-resistance genes/QTLs have been detected due to the development of molecular marker technology and methods for evaluating the resistance of rice to BPHs [2,3,22,23,24]. Among these 49 genes/QTLs, 33 (*Bph37* from IR64; *Bph38(t)*, *Bph33(t)*, *bph19*, *Bph31*, *Bph44(t)*, *qBph4.3*, *Bph33*, *Bph30*, *Bph41*, *Bph40*, *qBph4.1*, *Bph3*, and *qBph4.2* from IR65482-17, *qBph4.4*, *Bph17*, and *qBph4.2* from Rathu Heenati; *Bph27(t)*, *Bph6*, *Bph44*, *Bph42*, *Bph25*, and *Bph37* from SE382; *Bph32*, *bph4*, *Bph43*, *Bph28(t)*, *bph2*, *bph7*, and *Bph9* from Kaharamana; and *Bph1*, *Bph26*, and *Bph9* from Pokkali) were derived from traditional cultivated rice species; the rest were derived from wild rice varieties, including *Bph13(t)*, *bph11*, *qBph3*, *Bph14*, *qBph4,* and *Bph15* from *O. officinalis*; *Bph12* from *O. latifolia*; *Bph35*, *Bph36*, *Bph27*, and *bph29* from *O. rufipogon*; *Bph21* and *Bph20(t)* from *O. minuta*; *Bph34* from *O. nivara*; and *Bph18* and *Bph10* from *O. australiensis* (Table 1). Rice varieties containing one or more BPH-resistance genes/QTLs have been developed, and the cultivation of these varieties has greatly reduced the loss of rice yield induced by BPH feeding [25].

**Figure 1:**
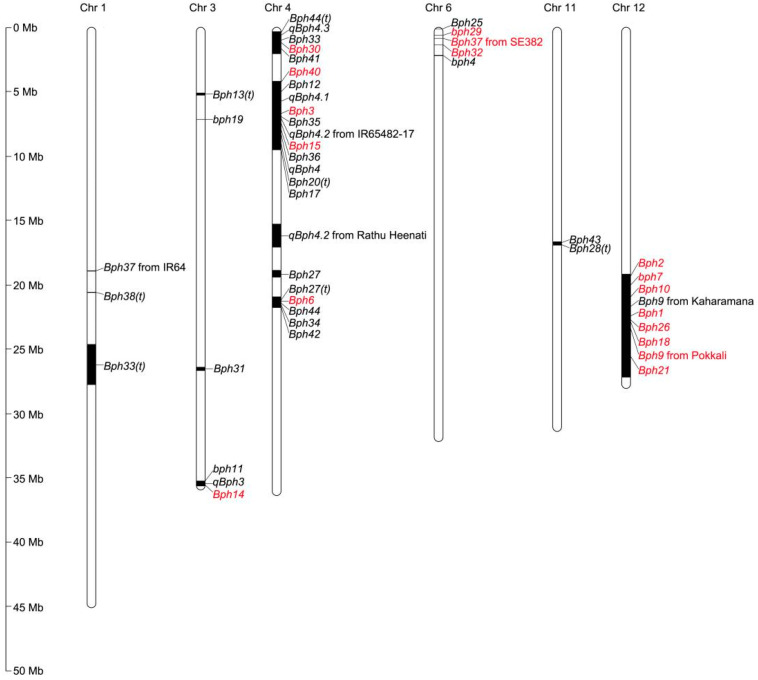
Distribution of BPH-resistance genes/QTLs on rice chromosomes. Numbers on the left indicate the physical distance. Black bars represented the position of BPH-resistance genes/QTLs on rice chromosomes. Red represents genes that have been cloned, and black represents genes that have not been cloned.

Most of the BPH-resistance genes/QTLs identified to date were located on six of twelve chromosomes (chromosomes 1, 3, 4, 6, 11, and 12), and their distribution on chromosomes was clustered (Figure 1). Three genes (*bph11*, *qBph3,* and *Bph14*) were clustered between 35.60 and 35.80 Mb of chromosome 3L [29,32,34]. A total of 21 genes were located on chromosome 4: five genes (*Bph44(t)*, *qBph4.3*, *Bph33*, *Bph30,* and *Bph41*) were clustered between 0.17 and 1.10 Mb on chromosome 4S [2,24,35,36,37]; 11 genes (*Bph40*, *Bph12*, *qBph4.1*, *Bph3*, *Bph35*, *qBph4.2*, *Bph15*, *Bph36*, *qBph4*, *Bph20(t),* and *Bph17*) were clustered between 4.44 and 9.38 Mb on chromosome 4S [2,3,33,38,39,40,41,42,43,44]; and *Bph27(t)*, *Bph6*, *Bph44*, *Bph34,* and *Bph42* were clustered on chromosome 4L between 20.60 and 21.80 Mb [24,37,45,46,47]. The *Bph37* that from SE382, *Bph25*, *bph29, Bph32* and *bph4* were present on chromosome 6S between 0.21 and 1.47 Mb [22,48,49,50,51]. The *Bph43* and *Bph28(t)* genes were clustered between 16.79 and 16.96 Mb of chromosome 11L [23,52]. Some regions of these genes in the same cluster might overlap, indicating that these genes were not the same but were tightly linked, or that they were the same gene. These clustered genes might also constitute different alleles of the same gene that mediate responses to different BPH populations. In the same region on chromosome 12L, a total of eight BPH-resistance genes have been isolated. Sequence alignment revealed that these genes were alleles, and four allelotypes were identified. An assessment of the BPH resistance of the four allelotypes revealed that the resistance to BPH populations conferred by allelotypes of the same resistance gene varies [54].

## 3. Cloning and Mechanisms of BPH-Resistance Genes

A total of 17 BPH-resistance genes have been isolated to date (Table 2). These genes can be classified into seven types based on the types of encoded proteins. Coiled-coil, nucleotide-binding, and leucine-rich repeat (CC–NB–LRR, CNL) protein is encoded by *Bph14* [34]. *Bph1*, *Bph2*, *Bph7*, *Bph9*, *Bph10*, *Bph18*, *Bph21*, *Bph26*, and *Bph37* encode atypical CC–NB–LRR proteins [22,54,59]. *Bph15* and *Bph3* encode lectin receptor-like kinases (LecRKs) [39,60]. Leucine-rich repeat domain (LRD)-containing proteins are encoded by *Bph30* and *Bph40* [2]. *Bph6* encodes an atypical LRR protein [46]. A B3 DNA-binding domain protein is encoded by *bph29* [49]. *Bph32* encodes a short consensus repeat (SCR) domain-containing protein [50]. The high variation in the types of proteins encoded by BPH-resistance genes reflected the high diversity in BPH-resistance mechanisms. These genes have been used to develop resistant rice varieties for the sustainable prevention and control of BPHs [25].

### 3.1. CC–NB–LRR Gene

*Bph14* was the first BPH-resistance gene to be cloned. This gene was isolated from the highly resistant line B5, which is a chromosome fragment infiltration line derived from the wild rice species *Oryza officinalis*. *Bph14* was first mapped on the long arm of chromosome 3 between markers R1925 and R2443 using the segregating population from the cross between B5 and Minghui 63 [3]. Du et al. fine-mapped *Bph14* and ultimately located it within the 34 kb interval between molecular markers SM1 and G1318 [34]. Two candidate genes, *Ra* and *Rb*, are present in this region. Transgenic functional verification revealed that *Ra* was *Bph14*. *Bph14* encodes a typical CNL protein, which is mainly accumulated in the vascular tissue. The salicylic acid (SA) signaling pathway was activated and callose deposition was induced in phloem during BPH feeding on plants expressing *Bph14* [34]. Additional studies have shown that BPH14 interacts with the transcription factors (TFs) OsWRKY46 and OsWRKY72 and activates the expression of the receptor-like cytoplasmic kinase gene *RLCK281* and the callose synthase gene *LOC_Os01g67364.1* in rice [23].

### 3.2. Atypical CC–NB–LRR Genes

A rare CC–NB–LRR protein with two NB domains (CC–NB–NB–LRR, CNNL) was encoded by *Bph9* [54]. Following BPH infestation, the SA signaling pathway was rapidly activated in plants expressing *Bph9* [54]. The CC domain of BPH9 has been shown to confer resistance to attack from BPHs. The NB1 and NB2 domains in BPH9 protein have been shown to be essential for the resistance of BPH9 to BPHs. NB2 domain with the intact NB function motifs repressed the activation of the CC domain. However, the NB1 domain did not have this function, as its sequence differed greatly from the NB function motifs. The LRR domain is responsible for the activation of BPH9 during BPH infestation [23]. *Bph9* was isolated from the *indica* rice variety Pokkali, and it was located in the interval on chromosome 12L between the markers InD2 and RsaI. The location of *Bph9* overlapped with the position intervals of seven other BPH-resistance genes (*Bph1*, *Bph2*, *Bph7*, *Bph10*, *Bph18*, *Bph21*, and *Bph26*). Genomic sequence alignment and analyses of the chromosomal locations of these genes have shown that the aforementioned eight genes were allelic to each other. Four allelotypes could be classified according to their sequences, and they conferred varying levels of resistance to three brown planthopper populations [54].

Another unusual CC–NB–LRR protein that lacked the LRR domain and only contained CC and NB domains was encoded by *Bph37* [22]. *Bph37* was mapped between 1.20 and 1.57 Mbp on chromosome 6S. In this region, a typical CC–NB–LRR protein was encoded by *LOC_Os06g03500* in the BPH-susceptible varieties Nipponbare and Kasalath. Whereas the premature termination of translation of *LOC_Os06g03500* in BPH-resistance variety SE382 was due to one base inserted in the second exon, which explained the absence of the LRR domain. Functional verification indicated that *LOC_Os06g03500* cloned from SE382 was *Bph37* [22]. The isolation of *Bph37* and studies of the domains of BPH14 and BPH9 suggest that the functions of the CC, NB, and LRR domains in BPH-resistance proteins may vary.

### 3.3. LRD Genes

Recently, the novel BPH-resistance gene *Bph30*, which encodes an LRD protein, was cloned from the cultivated rice variety AC-1613, and it was mainly expressed in the sclerenchyma cells of the rice leaf sheath [2]. BPH30 promotes the deposition of cellulose and hemicellulose in the sclerenchyma cell wall, which increases the cell wall stiffness and sclerenchyma thickness [2]. These structural changes impeded the ability of planthoppers to pierce the sclerenchyma with their stylets and feed on the phloem, thus conferring broad-spectrum resistance to planthoppers in rice [2]. Through the analysis of homologous genes and genome-wide association studies, the *Bph30*-like gene, *Bph40*, was isolated from the cultivated rice varieties SE232, SE67, and C334. *Bph40* encodes an LRD protein that was identified as BPH30. BPH40 has been shown to promote the deposition of cellulose and hemicellulose in the sclerenchyma cell wall, which might be similar to the resistance mechanism of BPH30 [2]. A total of 27 *Bph30*-like genes that encode proteins containing LRDs were identified in the Nipponbare genome. Whether other *Bph30*-like genes confer resistance to BPHs remains unclear. Future studies of *Bph30*-like genes may provide additional genetic resources and aid the development of more efficient strategies for isolating new BPH-resistance genes [2].

### 3.4. LecRK Genes

*Bph15* encodes an LecRK protein. This gene was derived from the resistant line B5, and it was initially mapped to a 0.4 cM interval on the short arm of chromosome 4 [3]. Next, a more refined genetic map was developed, and *Bph15* was located in a 47 kb interval between markers RG1 and RG2 [3]. *Bph15* from this region was isolated, and silencing this gene in rice weakened the anti-xenosis effect of BPHs [60]. *Bph3* was mapped to a 79 kb interval on the short arm of chromosome 4 [39]. The isolation and characterization of *Bph3* have indicated that *Bph3* is actually a cluster of genes encoding three lectin receptor-like kinases (OsLecRK1–OsLecRK3) [39]. Individual genes or a combination of two genes only confer partial resistance to BPHs, whereas the presence of all three genes confers durable and broad-spectrum resistance to BPHs and WBPHs in rice [39]. BPH15 and BPH3 are all localized to the plasma membrane, indicating that these four proteins might be pattern recognition receptors that receive herbivory-associated molecular patterns [23].

### 3.5. Other Types of BPH-Resistance Genes

*Bph6* encodes an atypical LRR protein [46]. This gene was initially mapped on the long arm of chromosome 4 between the simple sequence repeat (SSR) markers Y9 and Y19. It was derived from the Bangladesh landrace Swarnalata [3]. Guo et al. fine-mapped this gene and isolated it from the interval between the molecular markers H and Y9 [46]. BPH6 interacts with OsEXO70E1 and facilitates exocytosis [46]. *Bph6* confers broad-spectrum resistance to planthoppers by reinforcing the cell wall and activating SA, jasmonic acid (JA), and cytokinin (CK) signaling [46]. Recent studies have shown that BPH6 interacts with OsEXO70H3 and *S*-adenosylmethionine synthetase-like protein (SAMSL), which facilitates SAMSL secretion to the apoplast, where it promotes lignin deposition in the cell wall [61].

*bph29* is a recessive gene that was isolated from RBPH54. *bph29* was previously positioned on chromosome 6S between markers RM435 and RM540 [62]. Subsequent studies reduced the mapping range of *bph29* to 24 kb between markers BYL8 and BID2. A B3 DNA-binding domain protein is encoded by *bph29*. BPH infestation activates the SA signaling pathway, whereas it suppresses the JA/ethylene (ET) signaling pathway in RBPH54 [49]. *Bph32* was initially identified between markers RM19291 and RM8072 on the short arm of chromosome 6. This region was approximately 170 kb and 190 kb in 9311 and Nipponbare, respectively. Bioinformatics and DNA sequence comparison mediated the isolation of *Bph32* from Ptb33 [50]. *Bph32* encodes a protein with an SCR domain, and this protein confers resistance to BPHs by antibiosis [50].

## 4. Responses of Rice to BPH Infection

**Figure 2:**
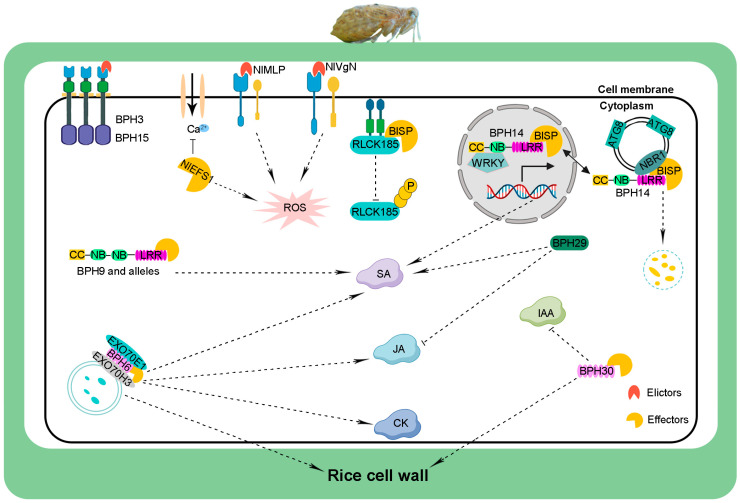
Model of rice-brown planthopper interactions. During BPH feeding on rice, elicitors and effectors were secreted into rice cells. Elicitors are perceived by PRRs, activating basic immune responses, such as elevated levels of ROS. However, effectors suppress the first-layer immune responses, such as BISP, which interacts with OsRLCK185 and suppresses its phosphorylation. The BPH-resistance proteins recognize these effectors, triggering second-layer immune reactions, including the activation or suppression of phytohormone signaling pathways and enhancement of the cell wall of leaf sheaths, thus inhibiting BPH from sucking phloem sap. ROS, reactive oxygen species; SA, salicylic acid; JA, jasmonic acid; CK, cytokinin; IAA, indole-3-acetic acid.

The host of BPHs started shifting from *Leersia* to rice approximately 0.25 million years ago [1]. BPHs then began to feed specifically on rice plants [3]. Rice plants have evolved multiple mechanisms to resist attack from BPHs (Figure 2) [2,3,46]. Several studies have focused on clarifying the molecular mechanisms of BPH resistance, and these studies have enhanced our understanding of the responses of rice to BPH feeding [3,46].

### 4.1. MAPK Signal Transduction

Mitogen-activated protein kinases (MAPKs) are a group of very conservative protein kinases in eukaryotes [63,64]. The activation of MAPKs is an early reaction of plant exposure to biotic and abiotic stress [63]. Biochemical and genetic studies have revealed that MAPK cascades connect the different stimuli and downstream responses in plants [23]. Several *OsMAPK* genes have been shown to alter the defense gene expression or phytohormone levels to regulate the resistance of rice to BPHs. *OsMAPK20-5* is a group D MAPK gene, and its expression was rapidly increased following female BPH infestation. BPH feeding increased the contents of ET and nitric oxide (NO) in *OsMAPK20-5* silencing plants, which increased the BPH resistance of rice [64]. *OsMKK3* was significantly induced after BPH infestation. The contents of JA, JA-Ile, and ABA were significantly increased, whereas the SA level was decreased in plants overexpressing *OsMKK3* during BPH feeding, thus compromising the preference for BPH feeding, survival rate, and reproduction [65]. Nanda et al. showed that the expression of *OsMPKs* was remarkably influenced by BPH population type, rice variety, and infestation period [66]. *OsSPL10* negatively regulated the resistance of rice against BPH. In *spl10* mutant plants, genes related to the MAPK signaling pathway were remarkably upregulated during BPH feeding [67]. NlDNAJB9 is a BPH salivary protein that is highly expressed in salivary glands. In the *NlDNAJB9* overexpression plants, MAPK cascades and other defense pathways were induced [68].

### 4.2. Phytohormones

Plant hormones play important roles in rice counteracting BPH. JA and SA are two of the most well-studied hormones involved in BPH resistance [69,70]. In *Bph14*-containing plants, BPH infestation increased SA content and the expression of SA-related genes, such as *EDS1*, *NPR1*, *ICS1*, *PAL*, and *PAD4* [34]. Similar changes have been observed in *Bph9*- or *bph29*-containing plants following BPH feeding [49,54]. Exogenous spraying of SA increased the resistance level of rice to BPHs, suggesting that SA positively regulated BPH resistance [46]. It is generally believed that JA and SA are two antagonistic plant hormones that play opposite roles in the resistance of rice to phloem-sucking insects [61]. However, this might not always be the case. In plants expressing *Bph6*, SA, and JA seemed to participate in the resistance in a synergistic manner [46]. Recent studies have shown that JA-deficient mutants are susceptible to BPHs, and SA deficiency has no effect on BPH resistance [70]. These findings indicate that the functions of JA and SA in the response of rice to attack from BPHs might vary with genotypes and genetic backgrounds.

CK, ET, gibberellins (GA), brassinosteroids (BR), abscisic acid (ABA), and indoleacetic-3-acid (IAA) were also related to the rice defense against BPHs. In *Bph6*-containing plants, the CK content and the expression of synthetic genes increased substantially between 12 h and 24 h following BPH feeding, and the BPH resistance of plants was significantly increased after treatment with CKs [46]. ET is a defense phytohormone that has multiple impacts on insect infestations. *OsACS2*, the 1-aminocyclopropane-1-carboxylic acid (ACC) synthase gene, plays a role in herbivore-induced ET biosynthesis in rice. Knockdown of *OsACS2* decreased the emission of ET and enhanced BPH resistance in rice [71]. The expression of *OsGID1*, a GA receptor gene in rice, was induced during BPH feeding. Overexpression of *OsGID1* improved the BPH resistance level of rice, which was attributed to the increase in the level of lignin and the upregulation expression of three SA pathway-related WRKY genes (*OsWRKY33*, *OsWRKY30*, and *OsWRKY13*) [72]. Exogenous spraying of BR activated JA pathways and suppressed SA pathways, which increased the susceptibility of rice to BPHs [73]. ABA is a key phytohormone that is not only involved in the regulation of plant development but also in the responses to stress. Exogenous treatment with ABA enhanced callose synthase activity but suppressed β-1,3-glucanase activity, which inhibited BPH feeding [74]. Recent studies have shown that IAA negatively regulates the BPH resistance of rice plants [75].

### 4.3. Transcription Factors

The defense responses of rice against BPHs are usually accompanied by the regulation of defense-related gene expression and defense-associated signaling transduction, and TFs play important roles in regulating these processes [76]. The expression of *OsWRKY45* was induced by BPH infestation and played a negative role in the BPH resistance of rice. In *OsWRKY45*-silenced plants, the content of H_2_O_2_ and ET was increased following BPH feeding, thus reducing the feeding, oviposition, and survival rate of BPH and delaying nymph development [77]. *OsWRKY53* positively regulated BPH resistance by increasing H_2_O_2_ production during BPH infestation [78]. *OsMYB30*, an R2R3 MYB TF, directly upregulated the expression of *OsPAL6* and *OsPAL8*, which encoded two key enzymes in the phenylalanine ammonia–lyase pathway and conferred BPH resistance in rice [69]. *OsERF3* encodes an ethylene-responsive factor that reduces the BPH resistance of rice, which might stem from the decrease in the BPH-elicited H_2_O_2_ content [79]. The microarray and RNA sequencing results revealed significant differences in both the number and expression of differentially expressed TFs in resistant and susceptible materials following BPH feeding [80].

### 4.4. Metabolites

Changes in large amounts of metabolites, including primary metabolites, secondary metabolites, and defense compounds, have been observed in rice following BPH infestation [34,75,81]. The contents of amino acids, which are the main metabolites in phloem sap and essential nutrients for BPHs, were significantly reduced in BPH-resistance rice varieties during BPH feeding [75]. This might motivate BPHs to seek BPH-sensitive materials to acquire more nutritious sap [75]. Lipid profiles of rice leaf sheaths showed that the sterol biosynthetic pathway in the susceptible variety Nipponbare and wax biosynthesis and phytol metabolism in resistant *Bph6*-transgenic plants were activated during BPH feeding [61]. A recent study showed that *Bph30* coordinated the flow of primary and secondary metabolites through the shikimate pathway, which conferred BPH resistance [75]. Serotonin is widespread in living organisms, and its synthesis was induced following BPH infestation. The suppression of serotonin biosynthesis increases levels of SA and enhances BPH resistance [81]. Schaftoside is a flavonoid that binds to the BPH CDK1 kinase NlCDK1 and affects its protein kinase activity, which reduces the survival of BPHs [82]. Callose is a well-studied compound involved in BPH resistance [23]. In BPH-resistant varieties, callose deposition blocked the phloem, which inhibited BPH feeding. In susceptible varieties, BPH infestation activated callose-hydrolyzing enzymes, which induced the degradation of callose and facilitated BPH feeding [34,46].

### 4.5. Calcium Signaling

Ca^2+^ is an important second messenger that is widespread in eukaryotes and plays a role in diverse biological processes [83]. Ca^2+^ influx was the earliest response of rice to BPH infestation [3]. *NlSEF1*, which is strongly expressed in the salivary glands of BPHs, encodes a Ca^2+^-binding protein that functions as an effector [84]. During BPH feeding, NlSEF1 is secreted into rice cells and decreases the cytosolic Ca^2+^ content, which is beneficial for the survival and feeding of BPHs [84]. This change in Ca^2+^ concentration is thought to function as a signal that elicits callose synthesis [61].

### 4.6. MicroRNAs

MicroRNAs (miRNAs) are single-stranded non-coding RNAs with a length of approximately 23 nt [85]. miRNAs bind target mRNAs through base complementary pairing to degrade them or inhibit translation, which mediates post-transcriptional gene silencing in both animals and plants [86]. Some studies have shown that miRNAs are involved in the responses of plants to external stimuli [87,88]. Wu et al. identified 23 miRNAs that were differentially expressed in *Bph15* introgression plants (P15) and the susceptible recipient line 9311 (PC) prior to BPH feeding [89]. A total of 104 and 80 differentially expressed miRNAs were identified in P15 and PC, respectively, following BPH infestation [89]. Significant differences in the abundance and expression levels of differentially expressed miRNAs in BPH-resistance and susceptible varieties before and after BPH feeding have also been identified in several other studies [90,91]. *OsmiR156*, the main regulatory factor of individual plant development, negatively regulates the BPH resistance of rice by increasing levels of JA [92]. Dai et al. found that *OsmiR396* silenced the expression of the *OsGRF8*, reducing the accumulation of transcripts of *OsF3H* and inhibiting flavonoid biosynthesis, thus negatively regulating BPH resistance in rice [93]. The results of these studies suggest that miRNAs play key roles in mediating the resistance of rice to BPHs. Additionally, these studies provided new target genes that could aid the breeding of BPH-resistance varieties.

## 5. BPH-Secreted Proteins That Involved in Rice–BPH Interactions

BPHs are typical piercing sucking insects that penetrate rice tissue with their stylets and suck phloem sap [94]. During the puncturing process, BPHs secrete a large number of proteins into rice tissues [95]. These secreted proteins are essential for the feeding success of BPHs and serve as key signaling molecules for initiating or suppressing rice immune responses (Figure 2) [96,97]. Several advances have been made in our understanding of BPH secretory proteins in recent years, and these studies have provided new insights into the interactions between rice and brown planthoppers [96,97,98].

### 5.1. BPH Elicitors

Elicitors are BPH secretory proteins that can be recognized by plants and trigger primary immune responses [99]. Mucin-like proteins are widespread in microorganisms [100]. *NlMLP* encodes an *N. lugens*-secreted mucin-like protein (NlMLP) identified from the BPH salivary glands [96]. During BPH feeding, NlMLP is secreted into rice tissues and induces rice defense responses, including the activation of the JA signaling pathway and MAP kinase, Ca^2+^ mobilization, and callose deposition [96]. NlMLP is indispensable for the assembly of stylet sheaths, and its silencing inhibits BPH feeding and performance [96]. Yolk proteins are crucial for egg development. The major precursors of yolk proteins, vitellogenins (Vgs), are usually cut into two segments [97]. NlVgN is the N-terminal subunit of the Vgs of BPHs and is present in saliva and eggs. The secretion of NlVgN into rice tissue induced direct defense responses, such as the production of JA-Ile, JA, cytosolic Ca^2+^, and H_2_O_2_, as well as indirect defense reactions, including the release of volatiles to attract female *A. nilaparvatae* wasps, which are natural enemies of BPHs. NlVg is also essential for the survival of BPHs, and disruptions in *NlVg* expression have a major effect on the feeding, development, and reproduction of BPHs [101]. *N. lugens* salivary protein 1 (NlSP1) was identified from the BPH salivary proteome. The secretion of NlSP1 into plants increases defense-related gene expression, H_2_O_2_ levels, and the deposition of callose [96].

### 5.2. BPH Effectors

Secretory proteins, known as effectors, can weaken defense responses [102,103]. *NlEG1* was identified in salivary glands and encoded an endo-β-1,4-glucanase with endoglucanase activity. The silencing of *NlEG1* reduced the ability of BPH stylets to puncture rice tissue. However, *NlEG1* silencing had no effect on the ability of BPHs to consume an artificial diet. NlEG1 did not induce defense-related responses following its secretion into rice tissues via BPH feeding. These findings suggest that NlEG1 functions as an effector that reduces the resistance conferred by the cell wall [104]. The flavone tricin is widespread in rice plants and can enhance the resistance of rice to BPHs. Gong et al. identified an effector, BPH salivary protein 7 (NlSP7), and found that it decreased the tricin level in rice, which promoted BPH feeding [98]. In addition to suppressing immune responses, effectors can be recognized by specific resistance proteins, activating more intense immune responses [102,105]. The effector BPH14-interacting salivary protein (BISP) was identified in a recent study. BISP interacts with OsRLCK185, which attenuates its autophosphorylation to suppress basal defense responses. The BPH-resistance protein BPH14 can bind to BISP to activate other resistance pathways to stop BPH feeding [106].

In addition to being able to activate or inhibit the defense responses of rice, the aforementioned secreted elicitors and effectors are also indispensable for the feeding, development, and reproduction of BPHs. Therefore, these proteins may become new targets for controlling BPH.

## 6. Conclusions and Future Perspectives

To date, more than 49 BPH-resistance genes/QTLs have been identified, and the utilization efficiency of these genes in the breeding of BPH-resistance varieties has been low; this has resulted in the homogeneity of BPH-resistance genes in rice varieties [25]. The application of these BPH-resistant varieties will impose enormous selection pressure on BPH to favor the evolution of mechanisms to overcome these resistance genes [107]. BPH populations capable of overcoming the defenses conferred by common BPH-resistance genes have been observed in the laboratory [108,109]. There are several explanations for the lack of utilization efficiency of these BPH-resistance genes. First, the resistance levels of BPH-resistance genes derived from different donors were greatly affected by genetic backgrounds and environments, and this induced significant variation in resistance in rice varieties containing the same resistance gene. Second, traditional approaches for introducing genes are time-consuming and laborious. Few germplasm resources containing different types of BPH-resistance genes and excellent agronomic traits have been developed. Consequently, donor parents with high BPH resistance and good agronomic traits are lacking. Third, the aforementioned resistance genes are mostly incompletely dominant, and the BPH resistance of heterozygous individuals was weaker than that of homozygous individuals [2]; thus, more labor and time will be required for BPH-resistance breeding, especially for hybrid rice breeding. Fourth, the BPH resistance mechanisms remain unclear, which impedes the cultivation of varieties with broad-spectrum resistance and the efficacy of gene pyramiding. However, the discovery of novel types of BPH-resistance genes and defense mechanisms has facilitated major advances in the breeding of BPH-resistance rice varieties [2,46]. A series of BPH-resistance varieties have been cultivated using traditional and molecular-assisted breeding approaches, and this has aided efforts to control BPH populations and enhance rice yield [25].

BPH-susceptible genes and miRNAs in rice merit increased attention, especially in light of the rapid development of genetic engineering technology [110,111]. To date, few BPH susceptibility genes have been identified. *OsERF3* plays a negative role in BPH resistance in rice by suppressing the biosynthesis of H_2_O_2_. The silencing of *OsERF3* enhanced the BPH resistance of rice [79]. *CYP71A1* is a cytochrome P450 gene. Knockout of *CYP71A1* in rice plants blocked serotonin synthesis but increased SA levels, which enhanced BPH resistance [81]. *OsACS2*, which encodes an ACC synthase, negatively regulates BPH resistance. The silencing of *OsACS2* increased the release of 2-heptanol and 2-heptanone, which are two repulsive volatiles, and suppressed BPHs infestation by attracting their natural enemy, *Anagrus nilaparvatae* [71]. Two miRNAs have also been identified to negatively regulate BPH resistance in rice [92,93]. Using genetic engineering methods to reshape the expression of these sensitive genetic materials, making BPH-susceptible varieties with excellent agronomic traits resistant is a fast and cost-effective breeding approach [112]. Although reprogramming these sensitive genetic materials may have deleterious effects on crop growth and yield, an improved understanding of BPH-resistance regulatory networks will greatly aid efforts to develop varieties that are resistant to BPHs without compromising yield [112].

Although studies of the mechanisms by which rice plants resist attack from BPHs can provide important insights that could aid the control of BPHs, studies of BPHs are also needed to achieve effective control of their populations. Many genomic, transcriptomic, and proteomic data on BPHs have been obtained in recent years, and several important elicitors and effectors have been discovered [96]. In addition to their roles in activating or inhibiting plant immunity, these proteins also play key roles in BPH feeding, and this merits increased research attention [96,97]. RNA interference can be used to silence target gene expression via double-stranded RNA (dsRNA) [113]. Although there are several challenges associated with the use of dsRNA, including its low stability in the environment, low absorption efficiency, and low intracellular delivery efficiency, dsRNA insecticides provide a green approach that could be effective for controlling infestations of insect pests [112,114,115,116,117]. This novel type of biopesticide has been used to control various diseases of plants in a sustainable and eco-friendly manner [118,119,120,121,122]. A system that co-delivers insecticide and dsRNA was recently developed that could be used to silence insecticide resistance genes in pests, which would increase the susceptibility of insect herbivores to insecticides [114,123,124,125]. This provides an optional approach for BPH control.

In addition, other environmentally friendly strategies for BPH management should also be considered. Cultural management is a simple and important eco-friendly method for controlling BPH, although it might not provide immediate results [107]. Crop rotation plays a significant role in restricting population of BPH. For instance, rotating rice with non-host plants or BPH-resistant varieties can remarkably decrease BPH populations [107]. Trap cropping is a useful strategy for BPH control and is commonly used in Asian countries [126]. Trap plants grow together with the major crop, attracting pests away from the major crop or attracting natural enemies, thereby protecting the main plants from pests [126,127,128]. One study showed that highly susceptible rice plants that were planted 20 d earlier around the main rice field attracted a large number of BPHs to feed, thus reducing the population on the major crop [126]. The pulling power of trap plants, planting time, and required space should be comprehensively considered before choosing trap crops [126]. Biological control, in which natural enemies such as predators and parasitoids play a major role, is another eco-friendly method for rectifying the harm of BPHs [107]. Predators of eggs, such as *Cyrtorhinus lividipennis*, larvae predators, including *Cyrtorhinus lividipennis*, *Lycosa. Pseudoannulata*, and *Migrovelia douglasi*, and predators of both larvae and adults, such as *Synharmoni octomaculata (F.)* and *Paederus fuscipes Curtis*, were found to significantly decrease BPH populations [129,130]. The parasitoids, such as *Oligosita yasumatsui*, *Anagrus* spp., and *Pseudogonatopus* spp. were also identified and considered indispensable factors in the biological control of BPHs [129,131].

In summary, the introduction of major BPH-resistance genes into rice varieties remains the most important method for BPH control. However, studies of the BPH resistance mechanisms of rice and improvements in genetic engineering technologies, as well as changes in agricultural practices will facilitate the development of a green tridimensional defense system to control BPH populations.

## Figures and Tables

**Table 1:** BPH-resistance genes/QTLs discovered in rice.

Gene	Germplasm	Chromosome	Linked Markers	Reference
*Bph37*	IR64	1L	RM302, YM35	[26]
*Bph38(t)*	Khazar	1L	693369, id1012165	[27]
*Bph33(t)*	RP2068	1L	RM488, RM11522	[28]
*Bph13(t)*	*O. officinalis*	3S	AJ09b, AJ09c	[29]
*bph19*	AS20-1	3S	RM6308, RM3134	[30]
*Bph31*	CR2711-76	3L	PA26, RM2334	[31]
*bph11*	*O. officinalis*	3L	G1318	[32]
*qBph3*	IR02W101 (*O. officinalis*)	3L	t6, f3, c3-14	[33]
*Bph14*	B5 (*O. officinalis*)	3L	SM1, G1318	[34]
*Bph44(t)*	IRGC 15344	4S	344-0-6, 344-1-2	[24]
*qBph4.3*	Salkathi	4S	RM551, RM335	[35]
*Bph33*	Kolayal, Poliyal	4S	H99, H101	[36]
*Bph30*	AC-1613	4S	SSR28, SSR69	[2]
*Bph41*	SWD10	4S	SWRm_01617, SWRm_01522	[37]
*Bph40*	SE232, SE67, C334	4S	-	[2]
*Bph12*	*O. latifolia*	4S	RM16459, RM1305	[3]
*qBph4.1*	Rathu Heenati	4S	-	[38]
*Bph3*	Rathu Heenati	4S	RHD9, RHC10	[39]
*Bph35*	RBPH660 (*O. rufipogon*)	4S	PSM16, R4M13	[40]
*qBph4.2*	IR65482-17	4S	RM261, S1, XC4-27	[41]
*Bph15*	B5 (*O. officinalis*)	4S	RG1, RG2	[3]
*qBph4.4*	Salkathi	4S	RM335, RM5633	[35]
*Bph36*	*O. rufipogon*	4L	S13, X48	[42]
*qBph4*	IR02W101 (*O.officinalis*)	4S	p17, xc4-27	[33]
*Bph20(t)*	*O. minuta*	4S	B42, B44	[43]
*Bph17*	Rathu Heenati	4S	RM8213, RM5953	[44]
*qBph4.2*	Rathu Heenati	4L	-	[38]
*Bph27*	*O. rufipogon*	4L	RM16766, RM17033	[42]
*Bph27(t)*	Balamawee	4L	Q52, Q20	[45]
*Bph6*	Swarnalata	4L	H, Y9	[46]
*Bph44*	Balamawee	4L	Q31, RM17007	[24]
*Bph34*	*O. nivara*	4L	RM16994, RM17007	[47]
*Bph42*	SWD10	4L	SWRm_01695, SWRm_00328	[37]
*Bph25*	ADR52	6S	S00310	[48]
*bph29*	*O. rufipogon*	6S	BYL8, BID2	[49]
*Bph37*	SE382	6S	-	[22]
*Bph32*	Ptb33	6S	RM19291, RM8072	[50]
*bph4*	Babawee	6S	RM190, C76A	[51]
*Bph43*	IRGC 8678	11L	16-22, 16-30	[23]
*Bph28(t)*	DV85	11L	Indel55, Indel66	[52]
*bph2*	ASD7	12L	RM7102, RM463	[53]
*bph7*	T12	12L	RM3448, RM313	[54]
*Bph10*	*O. australiensis*	12L	RG457	[55]
*Bph9*	Kaharamana	12L	RM463, RM5341	[56]
*Bph1*	Mudgo	12L	em5814N, em2802N	[57]
*Bph26*	ADR52	12L	DS72B4, DS173B	[58]
*Bph18*	*O. australiensis*	12L	BIM3, BN162	[59]
*Bph9*	Pokkali	12L	InD2, RsaI	[54]
*Bph21*	*O. minuta*	12L	S12094A, B122	[43]

S, short arm of chromosome; L, long arm of chromosome.

**Table 2:** BPH-resistance genes isolated from rice.

Gene	Germplasm	Chromosome	Encoded Protein	Defense Mechanism	Reference
*Bph14*	B5	3L	CC-NB-LRR	SA↑, callose deposition	[34]
*Bph9*	Pokkali	12L	CC-NB-NB-LRR	SA↑	[54]
*Bph1*	Mudgo	12L	CC-NB-NB-LRR	-	[54]
*Bph2*	ASD7	12L	CC-NB-NB-LRR	-	[54]
*bph7*	T12	12L	CC-NB-NB-LRR	-	[54]
*Bph10*	IR65482-4-136-2-2	12L	CC-NB-NB-LRR	-	[54]
*Bph18*	IR65482-7-216-1-2	12L	CC-NB-NB-LRR	-	[59]
*Bph21*	IR71033-121-15	12L	CC-NB-NB-LRR	-	[54]
*Bph26*	ADR52	12L	CC-NB-NB-LRR	-	[58]
*Bph37*	SE382	6S	CC-NB	-	[22]
*Bph6*	Swarnalata	4L	Atypical LRR	SA↑, JA↑, CK↑, enhanced cell walls	[46]
*Bph30*	AC-1613	4S	LRD	enhanced cell walls, IAA↓	[2]
*Bph40*	SE232, SE67, C334	4S	LRD	enhanced cell walls	[2]
*Bph15*	B5	4S	Lectin receptor kinase	*OsPR1a*↑, *OsLOX*↑, *OsCHS*↑	[60]
*Bph3*	Rathu Heenati	4S	Lectin receptor kinase	-	[39]
*bph29*	RBPH54	6S	B3 DNA-binding	SA↑, JA/ET↓	[49]
*Bph32*	PTB33	6S	SCR	-	[50]

S, short arm of chromosome; L, long arm of chromosome; CC, coiled coil domain; NB, nucleotide-binding domain; LRR, leucine-rich repeat domain; LRD, leucine-rich domain; SCR, short consensus repeat; SA, salicylic acid; JA, jasmonic acid; CK, cytokinin; IAA, indole-3-acetic acid; ET, ethylene; ↑, up-regulation; ↓, down-regulation.

## Data Availability

All data have been included in the manuscript.
